# Ferulic acid ameliorates nerve injury induced by cerebral ischemia in rats

**DOI:** 10.3892/etm.2014.2157

**Published:** 2014-12-24

**Authors:** LEIMING ZHANG, HONGSHENG WANG, TIAN WANG, NA JIANG, PENGFEI YU, YATING CHONG, FENGHUA FU

**Affiliations:** Department of Pharmacology, School of Pharmacy, Yantai University, Yantai, Shandong 264005, P.R. China

**Keywords:** ferulic acid, erythropoietin, cerebral ischemia, nerve injury

## Abstract

This study was designed to investigate the protective effect of ferulic acid (FA) on nerve injury induced by cerebral ischemia. Focal cerebral ischemia was induced by occlusion of the right middle cerebral artery and reperfusion 90 min later in male Sprague-Dawley rats. Daily treatment of the rats with FA was initiated 30 min after the surgery, and was continued for 7 days. The efficacy of FA against nerve injury was assessed by neurological deficit scores as well as pathohistological observation. The expression levels in the brain and level in the peripheral blood of erythropoietin (EPO) and granulocyte colony-stimulating factor (G-CSF) were analyzed by immunohistochemistry and enzyme-linked immunosorbent assay (ELISA), respectively. The results showed that FA attenuated nerve injury of the hippocampus, significantly ameliorated neurological deficits, and increased EPO but not G-CSF expression in the hippocampus and the peripheral blood of ischemic rats. The findings indicate that FA has certain protective effects on the nerve injury of cerebral ischemia, and suggest that promoting EPO in the brain and peripheral blood may be one of the neuroprotective mechanisms of FA.

## Introduction

Stroke is a serious threat to human health, and is recognized as the most important factor resulting in nerve injury (1,2). Evidence suggests that nerve injury occurs following ischemia in susceptible brain regions, such as the hippocampus and the cerebral cortex. Neuronal cell death has been identified by morphological analysis of the ischemic brain, and genetic and biochemical evidence further support the association of neuronal cell death with ischemia (3,4).

*Angelicae sinensis* is a traditional Chinese medicinal herb that has long been used to treat ischemic stroke and anemia (5,6). The chemical constituents of the extract of the roots of *Angelica sinensis* are classified into essential oil- and water-soluble materials (7). Ferulic acid (FA) is the main water-soluble component of the roots of *Angelica sinensis.* Previous studies have demonstrated that FA is able to enhance hematopoietic progenitor cell activity resulting in accelerated blood cell recovery by stimulating erythropoietin (EPO) and granulocyte colony-stimulating factor (G-CSF) expression (8,9). A further study demonstrated that FA was able to reduce cerebral infarction in a model of transient middle cerebral artery occlusion (10).

EPO and G-CSF were originally recognized as humoral mediators involved in the maturation and proliferation of hematopoietic progenitor cells (11), and their neuroprotective effects were subsequently identified. *In vitro* and *in vivo* studies in animal models revealed that exogenously administered EPO and G-CSF are neuroprotective (12,13). Endogenous EPO and G-CSF have also demonstrated beneficial effects in experimental stroke (14,15).

Although FA has been shown to reduce cerebral infarct secondary to transient focal cerebral ischemia, little data is available regarding its neuroprotective effect on nerve injury and the related protective factors EPO and G-CSF. Thus, the aim of this study was to investigate the protective effect of FA on nerve injury induced by cerebral ischemia and whether the cerebroprotective effect of FA is associated with EPO and G-CSF induction in the rat brain.

## Materials and methods

### Animals and drugs

Male Sprague-Dawley rats weighing 220±20 g were obtained from Experimental Animal Center of Shandong University of Chinese Traditional Medicine (Jinan, China). They were kept in air-conditioned rooms (temperature, 23±2°C) on a 12 h light-dark cycle, with free access to food and water. Animal experimental procedures were carried out in strict accordance with the guidelines published by the United States National Institutes of Health (NIH Publication no. 85-23, revised 1996) and approved by the ethics committee of Yantai University (Shandong, China). Surgical procedures, stroke induction and animal sacrifice (at the end of the observation period) were performed under general anesthesia with intraperitoneal (i.p.) injection of chloral hydrate (350 mg/kg).

The sodium salt of FA (i.e., sodium ferulate) was obtained from Haikou Qili Pharmaceutical Co., Ltd. (Haikou, China) and edaravone injection was provided by Nanjing Simcere Dongyuan Pharmaceutical Co., Ltd. (Nanjing, China).

### Rat cerebral ischemia study protocol

The middle cerebral artery occlusion (MCAO) procedure was carried out according to a previously described method with minor modifications (16). Briefly, rats were anesthetized with 10% chloral hydrate in 0.9% NaCl (350 mg/kg, i.p.) and placed in a dorsal recumbent position. Under sterile conditions, a ventral neck incision was made and the external carotid artery (ECA) and internal carotid artery (ICA) were exposed and carefully isolated. A nylon monofilament (40 mm in length and 0.25 mm in diameter), its tip rounded by flame-heating, was inserted from the lumen of the ECA to that of the right ICA to occlude the origin of the right MCA. The filament was removed after 1 h. For the sham group, the ECA and ICA underwent the same procedures without occlusion of the MCA. The rats were kept under conditions of controlled temperature (24–25°C) for the first 24 h after surgery.

The rats were randomly divided into six groups of 10 rats each. Three groups received different doses of FA (50, 100 and 200 mg/kg), and one group received edaravone (6 mg/kg) by intravenous injection 30 min after ischemia, and once a day thereafter for seven consecutive days. The rats in the sham and vehicle-treated group were injected with saline.

### Evaluation of neurological deficits

Neurological deficits were evaluated using a modified six-point scoring method (17), by an investigator who was blinded to each experimental group. The damage was graded on a scale of 0–5. The scale is: 0, no neurological deficits (normal); 1, failure to extend left forepaw fully (mild); 2, circling to the left (moderate); 3, falling to the left (severe); 4, no spontaneous walking with a depressed level of consciousness (very severe); and 5, death.

### Hematoxylin and eosin (H&E) staining

Six rats from each group were selected for H&E staining. The rats were deeply anesthetized with choral hydrate and pericardially perfused with 0.9% saline and then with 4% formaldehyde. The entire brain was embedded in paraffin. Nerve injury in the hippocampus was determined by analysis under a microscope (magnification, ×400; Olympus BX41; Olympus, Tokyo, Japan). The damage was evaluated by counting the number of surviving neurons per millimeter length of the hippocampus examined under light microscopy.

### Enzyme-linked immunosorbent assay (ELISA)

EPO and G-CSF levels in the plasma were measured by ELISA kits (EPO Quantikine ELISA Kit, catalogue no. MEP00B and G-CSF Quantikine ELISA Kit, catalogue no. MCS00; R&D Systems, Inc., Minneapolis, MN, USA). The rats in each group were sacrificed 24 h after the final administration of treatment and plasma was collected. Monoclonal antibodies specific for EPO and G-CSF were pre-coated onto microplates. Standards and samples were added to the wells and were incubated for 30 min at 37°C. After washing away the unbound substances, enzyme-linked polyclonal antibodies specific for EPO and G-CSF were added to the wells and were incubated for 60 min at 37°C. After removing any unbound antibodies, substrates were added to the wells and were incubated for 15 min at 37°C. The intensities of the colors developed were in proportion to the amount of EPO and G-CSF bound to the wells. The optical density of each well was measured with a scanning multi-well spectrophotometer (SpectraMax M3, Molecular Devices, Sunnyvale, CA, USA) at a wavelength of 450 nm.

### Immunohistochemistry assays

After being deeply anesthetized, the rats were transcardially perfused with saline solution, followed by 4% paraformaldehyde in 0.1 M phosphate-buffered saline (PBS) 24 h after ischemia. Brains were removed and post-fixed in 4% paraformaldehyde for 4 h, then transferred into 30% sucrose solution until the brains sank to the bottom of the container. Coronal sections (10 μm) were made using a Leica CM1950S cryostat (Leica Microsystems GmbH, Wetzlar, Germany). Sections were blocked with 3% normal goat serum (diluted in PBS containing 0.3% Triton X-100) for 1 h and incubated with rabbit anti-rat polyclonal primary antibodies [anti-EPO (BA0843) and anti-G-CSF (BA0746), 1:200, Wuhan Boster Biological Engineering Co., Ltd., Wuhan, China] overnight at 4°C. After rinsing with PBS, sections were incubated with horseradish peroxidase-conjugated goat anti-rabbit polyclonal IgG (ZDR-5306; Beijing Zhongshan Jinqiao Biological technology Co. Ltd., Beijing, China) as secondary antibodies (1:200) for 2 h at room temperature. The images from five fields of each ischemic region from six rats in each group were examined using the same brightness and exposure settings. Image-Pro Plus software (Media Cybernetics, Silver Spring, MD, USA) was used to analyze positive expression of EPO or G-CSF in each photograph. The artificial unit of mean optical density (MOD) × total per area (TPA) was employed for measurement in the stereological analysis, which indicated the integrated optical density (IOD) of the positive signal in the stained tissue.

### Statistical analysis

Neurological deficit scores between groups were analyzed using a non-parametric test. Quantitative data from the experiments were expressed as mean ± standard deviation (SD), and significance was determined by one-way analysis of variance (ANOVA) followed by Tukey’s test. In all cases, differences were considered significant if P<0.05.

## Results

### Effects of FA on neurological deficit scores in rats following MCAO

On the seventh day after the surgery, the results showed that FA induced a significant reduction in the neurological deficit score compared with that of the rats treated with vehicle. Treatment with FA at doses of 100 and 200 mg/kg but not 50 mg/kg significantly reduced the neurological deficit score in a dose-dependent manner in the MCAO model rats. Edaravone treatment, as a positive control, also significantly decreased the neurological deficit score (Table I).

### Effects of FA on neuronal damage in the hippocampus of rats following MCAO

**Figure 1:**
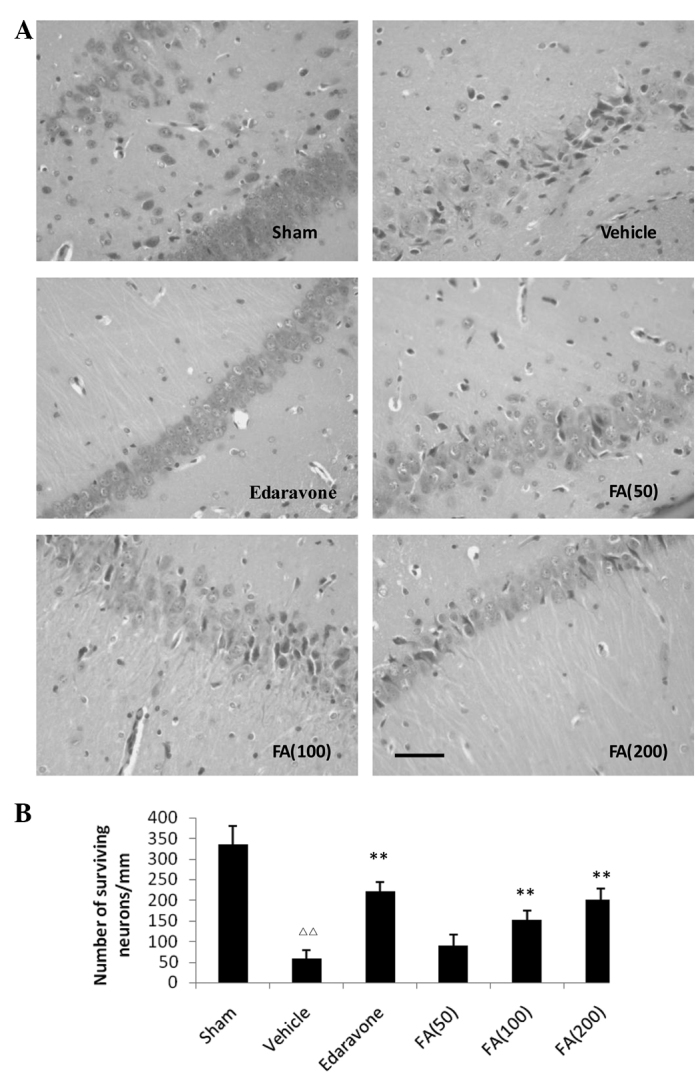
(A) Histopathology in the ischemic region following MCAO. Light microphotographs of the hippocampus with hematoxylin and eosin staining in the sham-treated control group, vehicle-treated ischemia model group, edaravone-treated group and FA-treated groups (50, 100 and 200 mg/kg). (B) Extensively damaged neurons in hippocampus were observed in the ischemia model group and the number of surviving neurons was significantly reduced compared with that in the sham-treated group. FA (at doses of 100 and 200 mg/kg) and edaravone treatment increased the number of neurons and numerous normal surviving neurons were observed. Scale bar: 40 μm; data expressed as mean ± standard deviation, n=6; ^ΔΔ^P<0.01 vs. sham-treated mice; ^**^P<0.01 vs. vehicle-treated ischemia model mice.

Extensively damaged neurons in the hippocampus were observed and the number of surviving neurons was significantly reduced in the vehicle-treated ischemic rats compared with the sham-treated rats. Neuronal shrinkage and chromatin condensation of nuclei were also observed in the ischemic rats. However, the number of surviving neurons in the FA-treated rats was higher compared with that in the vehicle-treated ischemic rats and numerous surviving neurons were observed in the hippocampus (Fig. 1).

### Effects of FA on EPO and G-CSF levels in the peripheral blood of rats following MCAO

FA induced significant enhancements in the EPO level in peripheral blood compared with that in the vehicle-treated rats (P<0.05). With regard to G-CSF levels, the rats treated with FA showed no significant difference compared with the vehicle-treated rats (Table II).

### Effects of FA on EPO and G-CSF expression in the hippocampus of rats following MCAO

**Figure 2:**
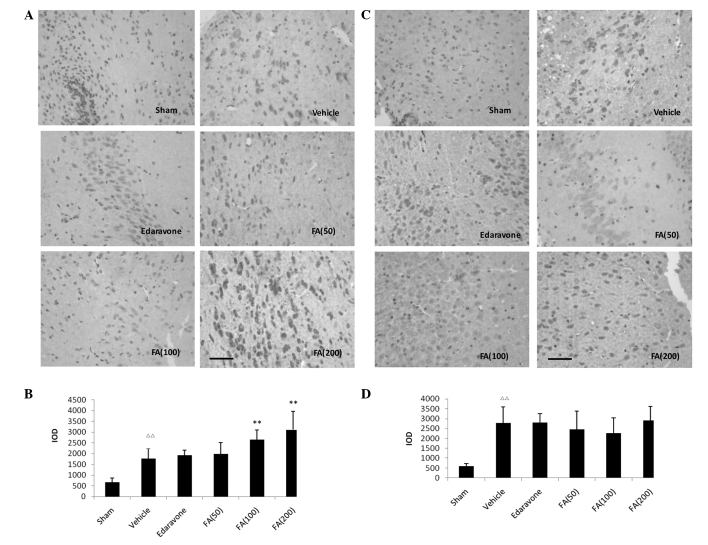
EPO and G-CSF expression as revealed by immunohistochemistry in rats following MCAO and the subsequent administration of different treatments. (A and B) EPO expression within the infarct region of the hippocampus in the vehicle-treated group was significantly increased compared with that of the sham-operated group. Administration of FA induced a significant enhancement in EPO expression in a dose-dependent manner (P<0.01). (C and D) In the case of G-CSF, cerebral ischemia induced a significant increase, while treatment with FA showed no significant difference in the expression of the protein in the infarct region, compared with that in the vehicle-treated rats. Scale bar: 40 μm; data expressed as mean ± standard deviation, n=6; ^ΔΔ^P<0.01 vs. sham-treated mice; ^**^P<0.01 vs. vehicle-treated ischemic mice. EPO, erythropoietin; G-CSF, granulocyte colony-stimulating factor; MCAO, middle cerebral artery occlusion; FA, ferulic acid.

The results showed that EPO expression within the infarct region in the vehicle-treated group was significantly increased compared with that of the sham-operated group. Administration of FA induced a significant enhancement in EPO expression levels in a dose-dependent manner (P<0.01). With regard to G-CSF, cerebral ischemia induced a significant increase in its expression, whereas treatment with FA was not observed to induce a significant difference in the expression of the protein in the infarct region compared with that in the vehicle-treated rats (Fig. 2).

## Discussion

Nerve injury resulting from focal or global cerebral ischemia is a major cause of mortality and disability in the adult population. Neuronal cell death has been observed to occur several days after ischemic insult and predominantly affects sensitive areas of the brain, such as the hippocampus and the cortex (18,19).

The results of the present study demonstrated that neuronal cell death occurred in the ischemic brain primarily in the hippocampal area seven days after ischemia. Treatment with FA significantly reduced the neurological deficit score and hippocampal neuron damage in a dose-dependent manner in the rats following MCAO. The results indicated that treatment of the rats with FA for seven days following MCAO ameliorated the neuronal damage.

EPO stimulates erythroid cell production, which supports the survival, proliferation and differentiation of erythroid progenitor cells. The sites of expression of the EPO receptor (EPO-R) and the EPO response are in hematopoietic cells and other cell types, including endothelial and neural cells (20). The targeted deletion of EPO or the EPO-R causes mice to lack definitive erythropoiesis and mature erythrocytes (21). In such mice, increased levels of apoptosis in the brain are also observed, which suggests that EPO, in addition to being necessary for the production of mature red blood cells, may also contribute to normal brain development (22). EPO is produced in fetal liver, adult kidney and also in the brain, in astrocytes and neurons; EPO production is induced by hypoxia, and may persist for 24 h or longer (23).

In the present study, it was observed that FA induced a significant enhancement of the level of EPO expression in the ischemic brain. The results also demonstrated that FA increased the EPO level in peripheral blood; EPO nay be transferred from the blood into the brain through the blood-brain barrier (BBB) (24). Therefore, the increasing levels of EPO in the brain and blood may contribute to the neuroprotective effect of FA.

The hematopoietic factor G-CSF was recently discovered to act as a protective and neurotrophic factor in the brain. Several studies have described the infarct-reducing and recovery-enhancing effects of G-CSF following ischemic stroke (25–27). The main actions of G-CSF are mediated via binding to the G-CSF receptors present on neuronal cells. In the present study, however, treatment with FA for seven days demonstrated no significant effect on the levels of G-CSF in the ischemic brain and peripheral blood. Although the traditional Chinese medicine *Angelicae sinensis* is commonly used to promote erythropoiesis for the treatment of anemia, and FA has previously been demonstrated to increase the levels of EPO and G-CSF (8,9), the results of the present study suggest that the neuroprotective effect of FA is not associated with G-CSF.

The findings indicate that FA has certain protective effects against the nerve injury induced by cerebral ischemia, and suggest that the promotion of EPO expression in the ischemic brain and peripheral blood may be one of the neuroprotective mechanisms of FA.

## Figures and Tables

**Table I:** Effects of FA on neurological deficit scores in rats following MCAO (n=10).

Group	Neurological deficit scores (median/range)
Sham	-
Vehicle	4/2
Edaravone	2/4^b^
FA (50 mg/kg)	4/4
FA (100 mg/kg)	3/4^a^
FA (200 mg/kg)	2/4^a^

FA, ferulic acid; MCAO, middle cerebral artery occlusion.

a P<0.05;

b P<0.01, compared with the vehicle group.

**Table II:** Effects of FA on EPO and G-CSF levels in the peripheral blood of rats following MCAO (mean ± standard deviation; n=6).

Group	EPO (μg/ml)	G-CSF (μg/ml)
Sham	8.32±2.85	0.39±0.09
Vehicle	8.63±0.97	0.78±0.24^c^
Edaravone	8.62±2.51	0.75±0.19
FA (50 mg/kg)	12.68±3.84^a^	0.62±0.11
FA (100 mg/kg)	13.28±3.32^a^	0.72±0.12
FA (200 mg/kg)	12.38±1.91^b^	0.60±0.12

FA, ferulic acid; EPO, erythropoietin; G-CSF, granulocyte colony-stimulating factor; MCAO, middle cerebral artery occlusion.

a P<0.05,

b P<0.01, compared with the vehicle group;

c P<0.01, compared with the sham group.
